# Retraction Note: Oroxylin A activates PKM1/HNF4 alpha to induce hepatoma differentiation and block cancer progression

**DOI:** 10.1038/s41419-025-07496-1

**Published:** 2025-03-11

**Authors:** Libin Wei, Yuanyuan Dai, Yuxin Zhou, Zihao He, Jingyue Yao, Li Zhao, Qinglong Guo, Lin Yang

**Affiliations:** State Key Laboratory of Natural Medicines, Jiangsu Key Laboratory of Carcinogenesis and Intervention, School of Basic Medicine and Clinical Pharmacy, China Pharmaceutical University, Nanjing, 24 Tongjiaxiang, People’s Republic of China

Retraction Note to: *Cell Death and Disease* 10.1038/cddis.2017.335, published online 20 July 2017

The Editors have retracted this article. Following publication, concerns have been identified with the figures in this article, specifically:

- In Figure 2a, the 96H [DMSO] panel appears to be a 180 degree rotation of the 72H [DMSO] panel.
- In Figure 2b, the SMMC-7721 beta-actin bands in the 96H treatment appear to be a flipped and widened version of the 72H treatment in the same panel; also the Hep G2 beta-actin bands in the 96H treatment appear to be a flipped and widened version of the 72H treatment in the same panel.
- In Figure 8f, the lower section of the HNF-4alpha [control] panel appears to be identical to the upper section of the HNF-4alpha [OA[75 mh/kg)] panel.

The Editors therefore no longer have confidence in the data.

Author Qinglong Guo does not agree to this retraction. Authors Libin Wei, Yuanyuan Dai, Yuxin Zhou, Zihao He, Jingyue Yao, Li Zhao and Lin Yang have not responded to correspondence from the editor or publisher about this retraction.

